# Estimating the prevalence of social and emotional loneliness across the adult lifespan

**DOI:** 10.1038/s41598-022-24084-x

**Published:** 2022-12-06

**Authors:** Aikaterini Manoli, Johanna McCarthy, Richard Ramsey

**Affiliations:** School of Psychological Sciences, Macquarie University, Level 3, Australian Hearing Hub, 16 University Ave, Sydney, NSW 2109 Australia

**Keywords:** Psychology, Health care, Public health

## Abstract

Loneliness is associated with detrimental consequences for mental and physical health. Even though loneliness affects people of all ages, very few studies have examined its prevalence across the adult lifespan. Additionally, no study has examined the distinction between social and emotional loneliness across the lifespan, even though it has long been considered functionally important. To address these issues, the present study examined the prevalence of social and emotional loneliness across the adult lifespan based on two cohorts (2016/17 and 2017/18) of a nationally representative survey (N ~ 8000 per cohort, age range: 16 to ~ 90). We estimated how similar or distinct patterns of social and emotional loneliness were across the adult lifespan and their consistency across cohorts. The results consistently showed that social and emotional loneliness levels differ as a function of age. Emotional loneliness peaked in younger and older adulthood, whereas social loneliness was stable in early and middle adulthood, before dropping steeply in later stages of life. These findings update basic understanding of loneliness by demonstrating how the experience of different loneliness types may vary across the adult lifespan. In the longer term, the findings have potential societal and clinical importance by informing interventions that target specific loneliness subtypes and age groups.

## Introduction

Humans are inherently social beings, whose lives are centered around interactions with others^1,2^. Due to the centrality of social life, the absence of sufficient social connections can be detrimental to our mental and physical well-being^3^. Indeed, loneliness, or a perceived lack of social connection^3,4^, has been associated with a range of negative health outcomes, such as higher immunodeficiency, susceptibility to psychiatric disorders, an increased risk for cognitive decline, and higher overall mortality^5–8^. As such, loneliness is an urgent public health issue, which requires further attention in order to mitigate its detrimental consequences.

Loneliness is often considered synonymous with social isolation, but these terms are dissociable^9^. Social isolation is an objective measure of the quantity of an individual’s social interactions, related to factors such as the frequency of social contact and proximity to others. In contrast, loneliness reflects a subjective perception that an individual’s need for social connection is not sufficiently being met by their surroundings^10–12^.

The prevalence of loneliness across the adult lifespan has received increased attention in recent years. Most studies have revealed a non-linear, U-shaped relationship between loneliness and age, whereby loneliness increases during young adulthood and older age, and substantially decreases in middle adulthood^3,13–18^. However, recent large-scale studies have also shown that loneliness has a linear relationship with age (i.e., loneliness gradually increases as people get older^19–21^), as well as a different nonlinear trajectory (i.e., loneliness decreases in young adulthood and gradually peaks from middle adulthood onwards^22^), or no relationship with age^23^. As such, even though a majority of prior studies have demonstrated a U-shaped trajectory of loneliness across the lifespan, the results remain mixed overall.

Although many factors could have contributed to these mixed findings (e.g., sociodemographic and health characteristics of the samples^20^), in this study we consider two factors that have not received sufficient attention in the research literature. First, the majority of studies have treated loneliness as a unidimensional concept, which could have masked relationships between age and different dimensions of loneliness. Loneliness is generally considered a multifaceted construct that is composed of at least two distinct components that have been identified for decades: social and emotional loneliness^24^. Social loneliness refers to the absence of a desired social network, that is, a wider circle of family, friends and acquaintances that can provide a sense of belonging^24–26^. In contrast, emotional loneliness is related to the lack of one or more attachment figures that one could form an intimate connection with^24–26^. While these two dimensions are correlated, they are distinct states that are associated with different events in someone’s life^27,28^. For example, social loneliness can occur after moving to a different location, whereas emotional loneliness can follow the death of a loved one^26^. Given that divergent accounts of the relationship between loneliness and age have been put forward^19,20,23^, it remains possible that each loneliness dimension has a distinct relationship with age.

A second factor that could have contributed to the variation in prior findings is that very few studies, if any, have investigated social and emotional loneliness across the entirety of the adult lifespan. For example, previous studies on young adults have found that emotional loneliness is more prevalent than social loneliness between adolescence and one’s late twenties^29,30^. Similar evidence was obtained in a sample of older adults (75+ years), in which emotional loneliness was more prevalent than social loneliness^26^. However, these prior studies focused only on younger or older adults, thus leaving middle adulthood understudied, and being unable to directly compare different loneliness types across the entire adult lifespan. Moreover, the loneliness measurements (i.e., social and emotional loneliness scale) and sample characteristics (e.g., nationality) in these studies are divergent, which makes comparison between results difficult, given that different national backgrounds and loneliness measures can influence loneliness scores^20,31,32^. Therefore, the prevalence of social and emotional loneliness across the adult lifespan remains unclear.

In light of this research context, the current project investigates the severity of social and emotional loneliness across the adult lifespan. We use a nationally representative sample of adults aged 16 to ~ 90 to estimate the relationship between age and each of the two loneliness dimensions. More specifically, we use data from two different years of the National Survey for Wales (NSW), an annual survey of approximately 10,000 people in Wales, which included a loneliness questionnaire. We take a multi-level Bayesian estimation approach to analyse the data over two cohorts (2016/17 and 2017/18). As such, we use a data-driven approach to test the extent to which patterns of social and emotional loneliness are similar or distinct across the adult lifespan, as well as how consistent the data are across two large cohorts that cover the entirety of the adult lifespan. We also evaluate the extent to which levels of social and emotional loneliness across the adult lifespan support the differing predictions from previously suggested trajectories of loneliness across the lifespan. The primary value of the current work is to improve basic understanding of the rate of social and emotional loneliness in different stages of adult life. In the longer term, such findings could also ultimately help to inform interventions targeting specific loneliness symptoms across the lifespan.

## Materials and methods

### Data source

The present study involved secondary analyses of two existing datasets from the 2016/17 and 2017/18 cohorts of the NSW, an annual survey conducted by the UK's Office of National Statistics (ONS). The NSW is a nationally representative sample of the entire adult population (aged 15 and over) of Wales, assessing factors like health status, lifestyle, access to public services, mental wellbeing and loneliness. The design of the NSW is described in more detail in the Welsh Government Technical Report^33^. In total, there were 21,874 participants across both NSW datasets (10,493 in the 2016/17 dataset and 11,381 in the 2017/18 dataset). Participants in each cohort were randomly chosen to participate from the entire population of Wales. Only participants who completed the loneliness measure in the NSW were included in the study. Thus, the final sample consisted of 8177 individuals (4559 females) from the 2016/17 dataset and 9343 individuals (5,168 females) from the 2018/19 dataset. The age range of participants in the 2016/17 and 2018/19 datasets was 15 to 100 years (M = 55.16, SD = 18.38) and 15 to 90 years (M = 54.2, SD = 18.23), respectively. Age was treated as a continuous variable in this study. In both datasets, over 95% of participants identified as Caucasian (Welsh or British). Additional demographic information for each dataset can be found in Supplementary Table S1.

### Social and emotional loneliness measure

Loneliness was measured by the 6-item De Jong-Gierveld Loneliness Scale (DJGLS), which is a shortened version of the 11-item DJGLS^34^. The 6-item DJGLS contains two 3-item subscales that measure social (e.g., “There are plenty of people I can rely on when I have problems”) and emotional loneliness (e.g., “I experience a general sense of emptiness”). Each item is answered categorically (“Yes” / “More or less” / “No”). This item response model is based on dichotomous item scale scores in which the answer “more or less” always indicates loneliness^31^. This means, for example, that answers of “yes” or “more or less” are given a score of 1, while answers of “no” are scored as 0. Responses in each subscale are summed to produce a score from 0 to 3, with greater scores indicating greater social or emotional loneliness.

The validity and reliability of the 6-item DJGLS have been empirically tested for use in different age groups and cultures^31,34^. The 6-item scale has been found to have good internal consistency (α reliability coefficients between .70 and .76) and strong correlations (*r* correlation coefficients between .93 and .95) with the original 11-item DJGLS, indicating good convergent validity^34^. The emotional and social subscales have good reliability (α reliability coefficients between .82 to .90 for emotional and α between .85 to .94 for social loneliness)^34^. Moreover, confirmatory factor analysis has confirmed the existence of each type of loneliness as separate constructs^34^.

### Data analysis approach

We followed a Bayesian estimation approach to modelling^35^, which we have adopted in recent papers^36,37^. The main goal was to estimate parameters of interest in regression models of varying complexity and compare the performance of these models. First, we evaluated key parameters of interest from the posterior distribution of our most complex model. Second, we performed model comparison via efficient approximate leave-one-out cross validation (LOO^38^), to estimate how accurately each model can predict out-of-sample data. In this way, we could estimate the extent to which an increase in model complexity corresponded to an increase in model accuracy.

More specifically, we implemented McElreath’s^35^ general Bayesian principles in the modelling package “brms”^39–41^ in R version 4.1.1^42^. Given that our primary dependent variable (loneliness score) was a count variable, we modelled the data using a binomial distribution. We computed six models per dataset (2016/17, 2017/18), which built incrementally in terms of complexity. We started with an intercepts-only model (m0). Model m1 included a linear age term and model m1b also included a quadratic age term. Model m2 additionally included loneliness type, and model m3 included a linear age * loneliness type interaction term. Lastly, model m3b additionally included a quadratic age * loneliness type interaction term. Model m3b, therefore, was the full or most complex model in each dataset.

Factors were coded according to a deviation coding style, where factors sum to zero. As such, loneliness type was coded as −0.5 (social) and 0.5 (emotional). Age was centred and standardised. We set priors using a weakly informative approach^43^, because we currently do not have sufficient knowledge to place more specific constraints on expected results (Table 1). Weakly informative priors place a constrained distribution on expected results, rather than leaving all results to be equally likely (uniform). Also, by using weakly informative priors, we allow for the possibility of larger effects, should they exist in the data^43–46^. Given the relatively small effect sizes in the field of psychology in general^47^, we centred normally distributed priors for effects of interest on zero (i.e., no effect) with a standard deviation of 0.5 (see class “b” in Table 1). That means that prior to running the study, we expected effects closer to zero to be more likely than effects further away from zero.

**Table 1:** Weakly informative priors.

Prior	Class
Normal (0, 1)	Intercept
Normal (0, 0.5)	b

b = population-level or fixed effects.

The formula for the full model (m3b) is specified below, with *l*_*count*_ being the dependent variable, *age* being the linear age term, *age*^2^ being the quadratic age term and *l*_*type*_ being the loneliness type factor (social vs. emotional):

$$l_{count} \sim 1 | trials\left( 3 \right) \sim 1 + age*l_{type} + age^{2} *l_{type}$$

For all six models (m0 to m3b) we subsequently computed LOO to compare out-of-sample prediction accuracy. Lastly, to follow-up our primary analyses, we also ran some further, more exploratory analyses. Given that other demographic and social contact variables have been associated with loneliness in the NSW, such as marital status, health and socioeconomic status (e.g.^10^), we wanted to run a further model that included a set of additional covariates. By doing so, we wanted to see the extent to which our key parameters of interest changed once additional covariates were added. Therefore, we ran one further model, which expanded model m3b to include five further variables including gender, marital status, number of people in a household, a measure of deprivation and a measure of general health (see Supplementary Materials for details).

## Results

### Describing social and emotional loneliness across the lifespan

**Figure 1:**
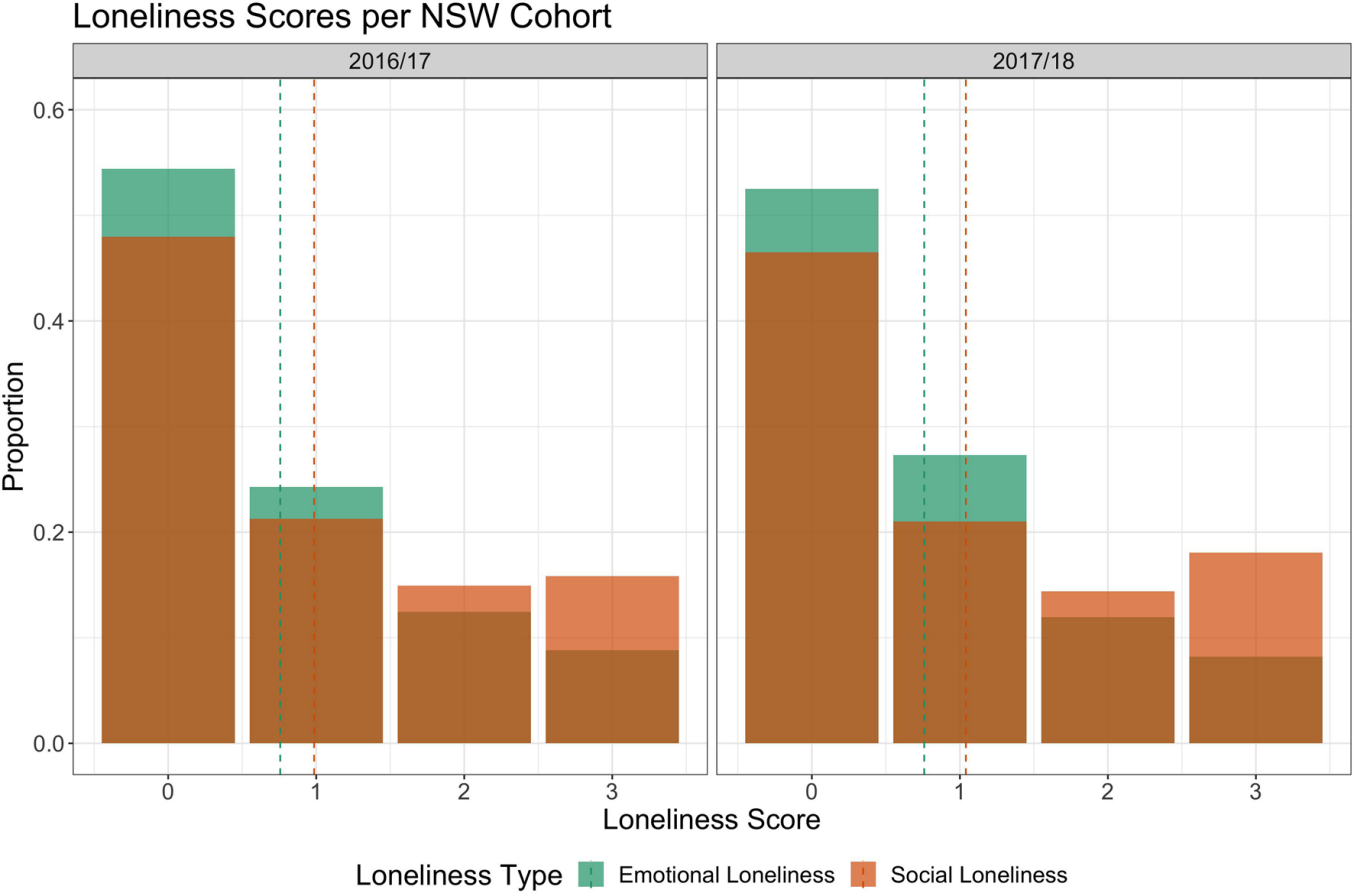
Social and emotional loneliness scores in the two NSW cohorts (2016/17 and 2017/18) (note: bars for social and emotional loneliness are overlapping). Scores for each loneliness type range between 0 (no loneliness) and 3 (severe loneliness). Dashed lines represent mean scores for each loneliness type.

**Figure 2:**
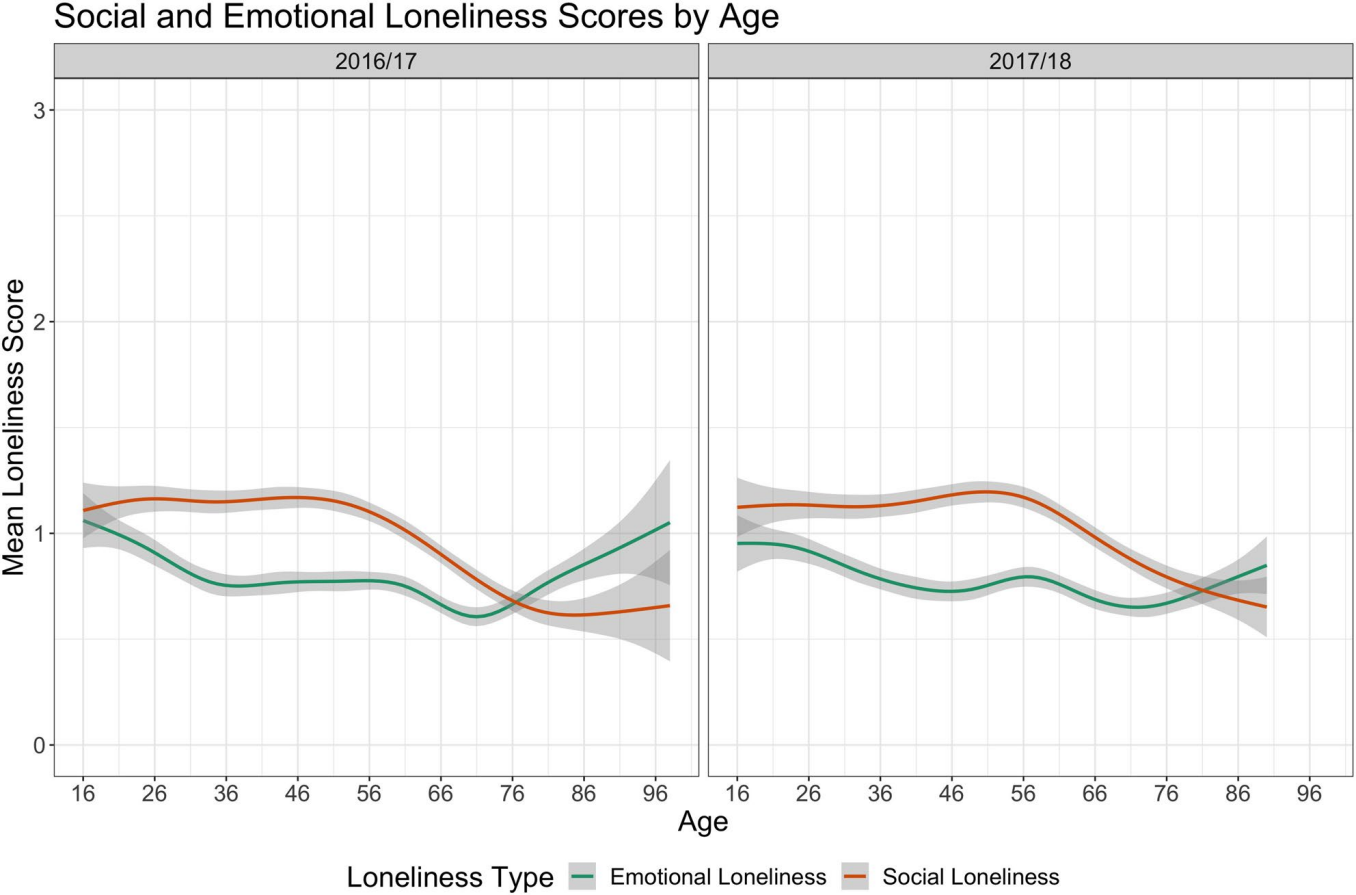
Mean social and emotional loneliness scores across the adult lifespan in the two NSW cohorts (2016/17 and 2017/18), along with 95% confidence intervals for each loneliness type. Scores for each loneliness type range between 0 (no loneliness) to 3 (severe loneliness).

Our primary aim was to investigate the extent to which levels of social and emotional loneliness are similar or distinct across the adult lifespan in two nationally representative cohorts. Social and emotional loneliness scores were largely consistent between the two cohorts (Figs. 1 and 2). Figure 1 shows social and emotional loneliness scores in the two NSW datasets. Most participants reported not experiencing loneliness or low levels of loneliness in both cohorts. Mean scores for social loneliness were 1.04 (SD = 1.15) and 0.99 (SD = 1.12) for the 2016/17 and 2017/18 cohorts, respectively. Additionally, mean scores for emotional loneliness were 0.76 (SD = 0.96) and 0.77 (SD = 0.98) for the 2016/17 and 2017/18 datasets, respectively.

Figure 2 shows the mean social and emotional loneliness scores across the lifespan. Social and emotional loneliness had a different distribution across age. Social loneliness remained relatively stable in early and mid-adulthood and dropped in late adulthood (between 56 and 96 years of age). Emotional loneliness followed a subtle U-shaped distribution, peaking in early and late adulthood, and dropping between 26 and 86 years of age. In addition, social loneliness was higher than emotional loneliness until late adulthood (~ 80 years of age in each cohort), at which point emotional loneliness increased.

### Assessing the levels of social and emotional loneliness across the adult lifespan

**Figure 3:**
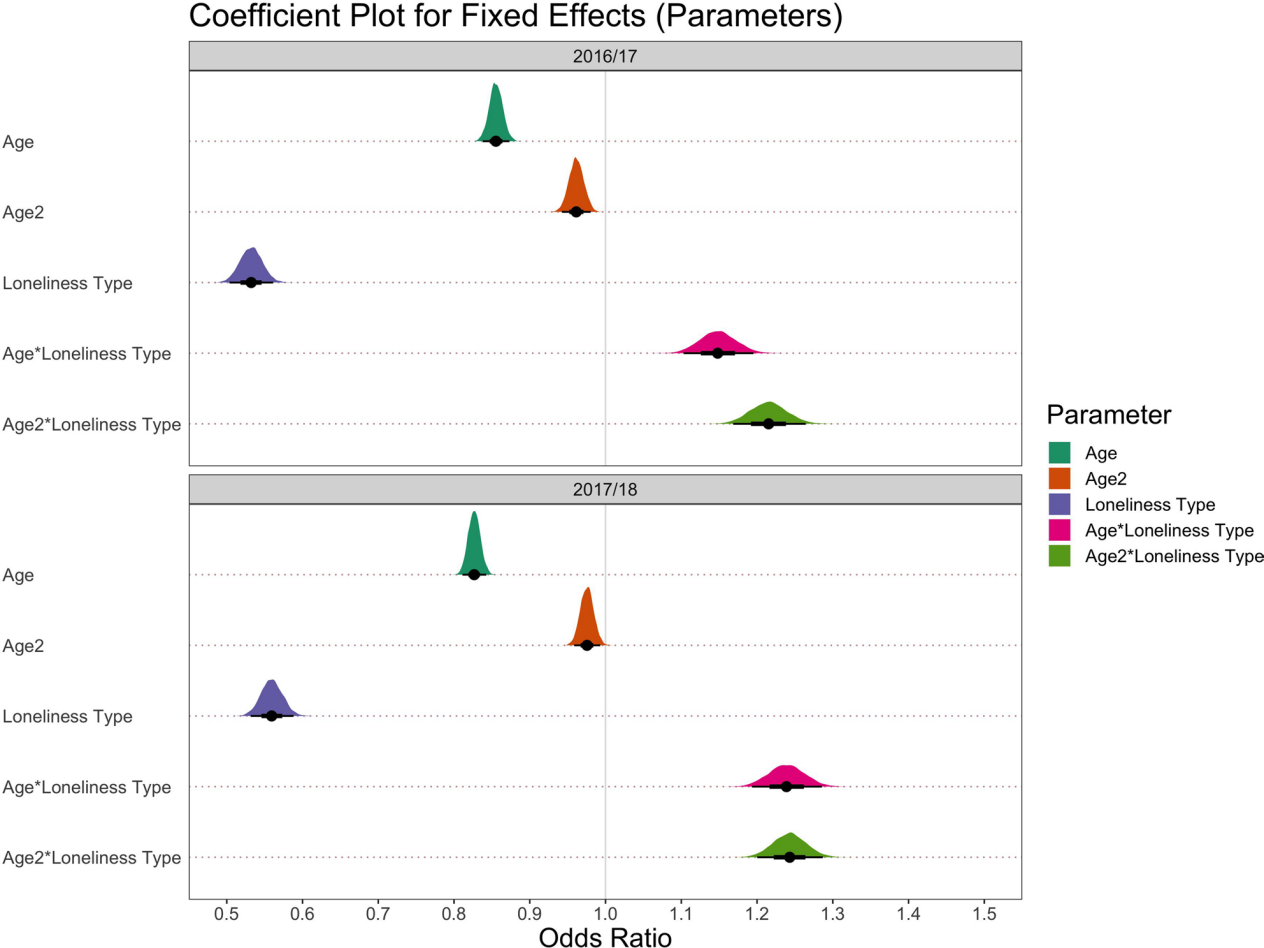
Odds ratios (fixed effects) of parameters for the most complex models (m3b) in each NSW dataset (2016/17, 2017/18).

We assessed the levels of loneliness types across the adult lifespan in two ways. First, we considered parameter estimates in the most complex of our primary models. For both sets of analyses (2016/17 and 2017/18 NSW cohorts), model diagnostics were acceptable, and the chains converged well, which means that the models were built without any problems (see Supplementary Figs. 1–4). Additionally, for both analyses, parameter estimates for the most complex model (m3b) are shown in Fig. 3 and Table 2. Parameter estimates were converted from log odds to odds ratios for ease of interpretation. An odds ratio of 1 indicates no effect on the outcome, while an odds ratio of 1.5 indicates a 50% increase in the outcome measure.

**Table 2:** Odds ratios of parameters for the full models (m3b) in the 2016/17 and 2017/18 NSW cohorts.

Year	Parameter	Odds ratio	Lower QI	Upper QI
2016/17	Age	0.86	0.84	0.87
	Age^2^	0.96	0.94	0.98
	Loneliness type	0.53	0.50	0.56
	Age * Loneliness type	1.15	1.10	1.20
	Age^2^ * Loneliness type	1.22	1.17	1.26
2017/18	Age	0.83	0.81	0.84
	Age^2^	0.98	0.96	0.99
	Loneliness type	0.56	0.53	0.59
	Age * Loneliness type	1.24	1.19	1.29
	Age^2^ * Loneliness type	1.24	1.20	1.29

Lower QI = lower bound of the 95% Bayesian quantile interval; upper QI = upper bound of the 95% Bayesian quantile interval.

Results across both years of the NSW were consistent. The odds ratios for the fixed effects of linear age, quadratic age, and loneliness type were negative, with the lower bound of the interval estimate ranging from 0.8 to 0.9 for linear and quadratic age terms, and 0.5 for loneliness type. These main effects are qualified by the interaction terms, which are directly relevant to our primary aim. The odds ratio for the interactions between both age terms (linear and quadratic) and loneliness type was positive with the lower bound of the interval estimate ranging from 1.1 to 1.2 (Fig. 3, Table 2). This suggests that the shape of the linear and quadratic age effects varied as a function of loneliness type. In other words, the relationship between each loneliness type and age was not the same.

**Figure 4:**
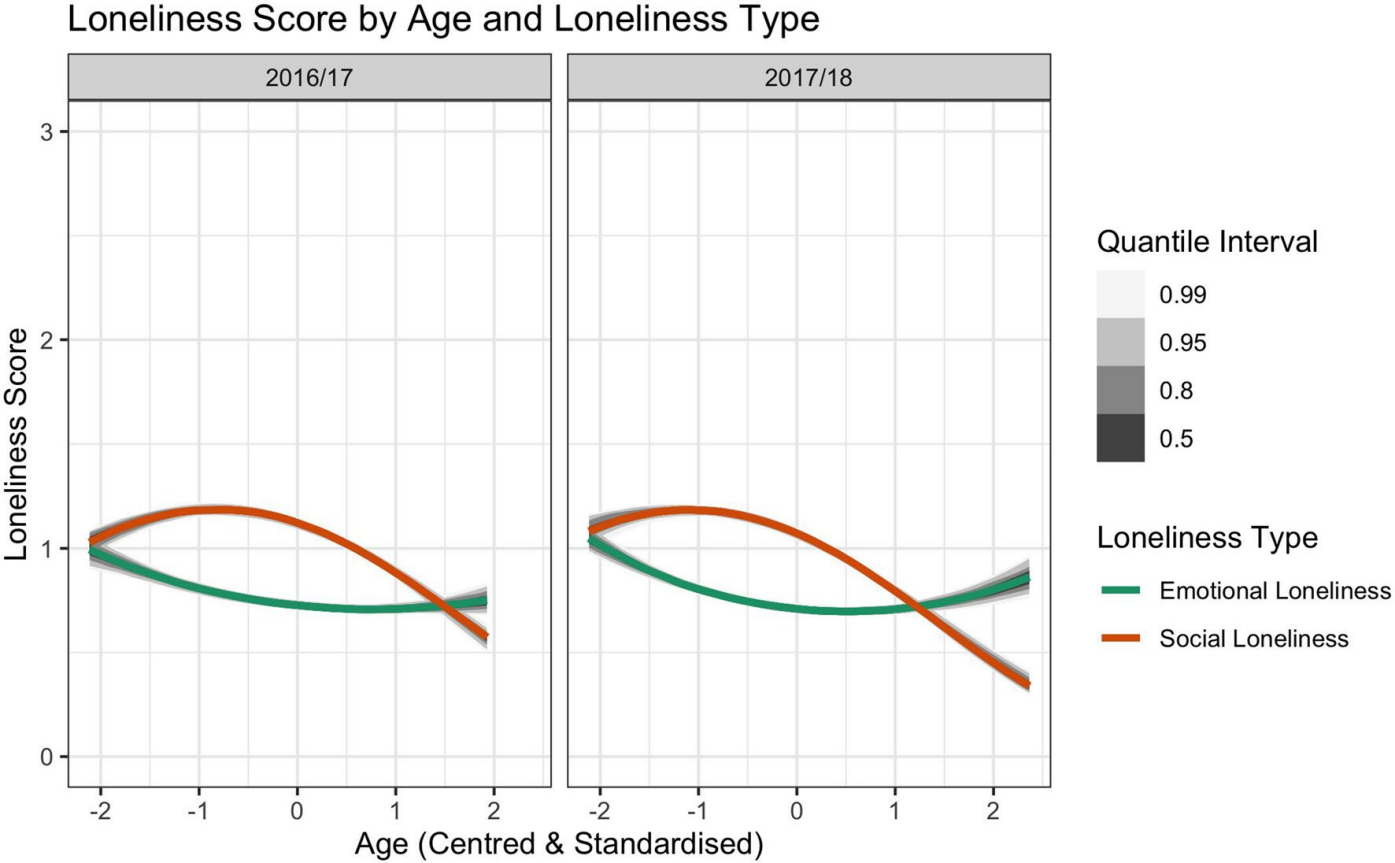
Predictions for social and emotional loneliness scores across the lifespan based on the full model (m3b) outputs for each NSW cohort (2016/17, 2017/18), along with 50%, 80%, 95%, and 99% quantile intervals.

According to the model predictions (Fig. 4), the pattern of social loneliness across age was steeper and curvier than the pattern of emotional loneliness. Therefore, given that social loneliness was coded as a negative factor (− 0.5) and emotional loneliness as a positive factor (+ 0.5), a positive interaction term indicates that the linear and quadratic age effects were more positive for emotional than social loneliness. This interaction effect can be readily seen in Fig. 4, whereby emotional loneliness is well-characterised by a relatively gentle U-shaped function across age. That is, emotional loneliness peaks in the youngest and oldest cohorts. In contrast, social loneliness remains relatively stable in the first half of the age cohort (although higher than emotional loneliness), and then reduces in the second half of the age cohort. The centre point (i.e., zero) in Fig. 4 resembles the mean age in each cohort, which was 54 and 55 years for 2016/17 and 2017/18, respectively.

**Figure 5:**
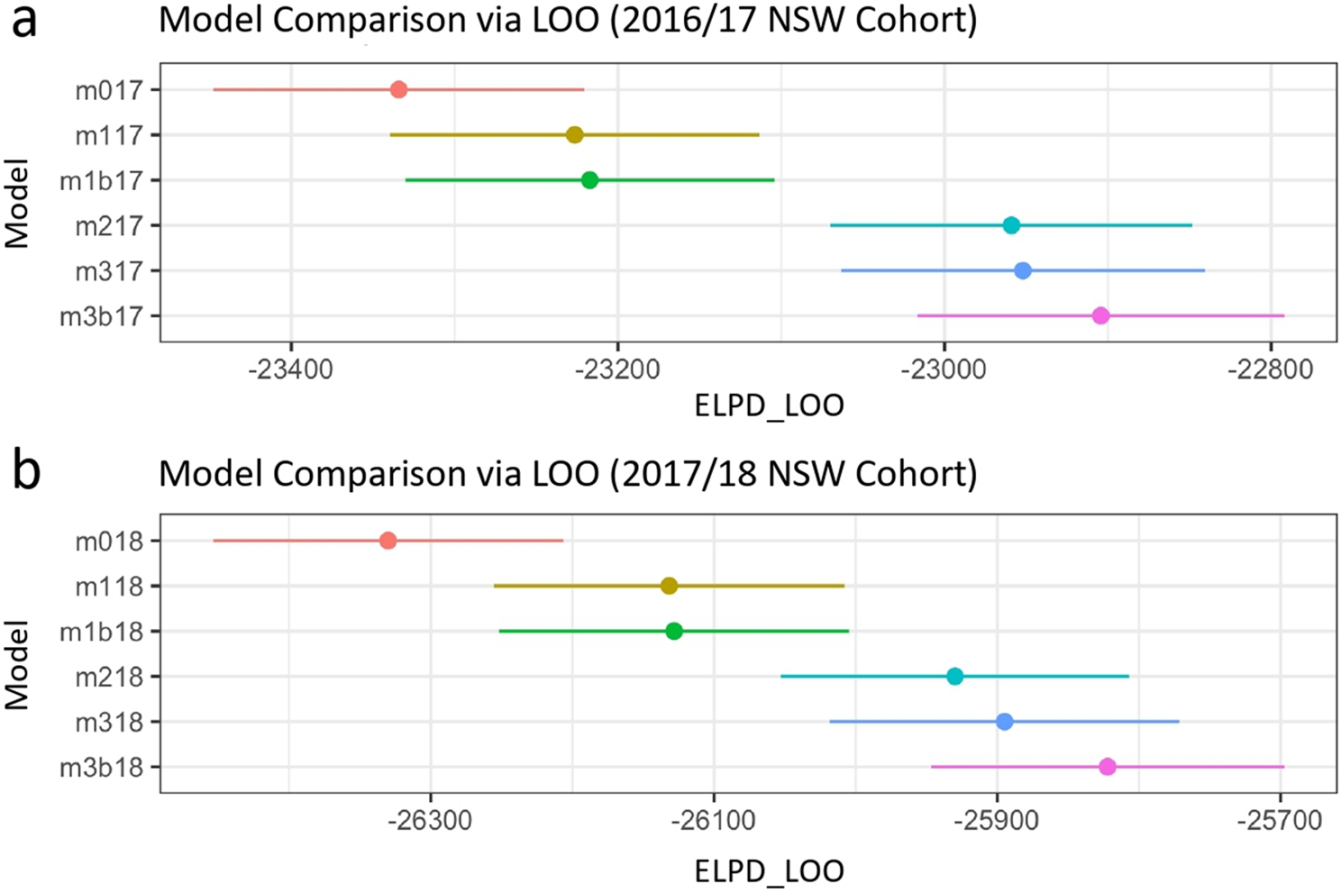
Model comparison (models 1-6) for 2016/17 (**a**) and 2017/18 (**b**) NSW cohorts. In both analyses, model m0 included an overall intercept; model m1 added a linear age term; model m1b added a quadratic age term; model m2 added a predictor for loneliness type (social vs emotional); model m3 added a linear age * loneliness type interaction; model m3b (the full model) added a quadratic age * loneliness type interaction. “ELPD_LOO” refers to the estimate of the expected log pointwise predictive density. “LOO” refers to leave-one-out estimated cross validation. Error bars depict the standard error of the mean. The “17” and “18” suffixes in each model indicate the 2016/17 and 2017/18 NSW cohorts, respectively.

We also ran an additional exploratory model with factors that are frequently associated with loneliness as covariates (i.e., gender, marital status, number of people in a household, a measure of deprivation and a measure of general health). Once these covariates were added in, the primary results, which involved interaction terms between linear and quadratic age terms and loneliness type, remained the same in terms of strength and direction (Supplementary Figs. 5–6, Supplementary Table S2). That is, there was still a clear positive interaction term for each age term and loneliness type. We take this finding as suggestive evidence that whatever set of variables contribute to the relationship between age and loneliness type, it cannot be easily described by a host of demographic, health and social contact variables that have previously been associated with loneliness.

The second way we assessed loneliness across the adult lifespan was by comparing out-of-sample predictive accuracy across models. The pattern of predictive accuracy across models was consistent across both datasets (Fig. 5). Compared to the intercepts-only model (m0), adding loneliness type into the model (m2) improved out-of-sample predictive accuracy more than adding either age term (m1 and m1b). In fact, error bars for age models overlapped with the intercepts-only model, which suggests that they were largely similar. Likewise, error bars for the models with the interaction terms (m3 and m3b) also overlapped with the model with loneliness type (m2), which again suggests that these models performed in a largely similar manner in terms of out-of-sample predictive accuracy.

## Discussion

This is the first large-scale study to investigate the levels of social and emotional loneliness across the adult lifespan. Across both datasets, the results demonstrated a consistent relationship between loneliness type and age. In both datasets, levels of social loneliness peaked in early and middle adulthood, and dropped in later adulthood. In contrast, emotional loneliness followed a gentle U-shaped distribution, peaking in early and late adulthood, and dropping in middle adulthood. These results provide large-scale empirical evidence that demonstrates how levels of social and emotional loneliness, which have long-been considered functionally important for individuals and society^24^, vary across the adult lifespan. In the following, we consider implications for understanding the prevalence of loneliness in society, as well as for designing future interventions, and we also highlight limitations of the current work.

### Comparison of results to previous models of loneliness and age

We first consider how our findings fit with the predictions from prior models of loneliness. The distribution of emotional loneliness supports a non-linear trajectory of the relationship between loneliness and age^3,13,14,21^. In line with previous findings, emotional loneliness was at its highest points in young and older adulthood^26,29,30^. Although the cross-sectional nature of the current design is unable to demonstrate which factors are driving such a relationship, the findings are intuitively consistent with past work, which has associated emotional loneliness with the presence of an intimate partner^26,29,48,49^. As such, emotional loneliness would be higher before intimate relationships are established (younger age) and once they have ended (older age), for example due to divorce or a partner’s death.

Social loneliness, however, did not follow predictions from prior models. For example, social loneliness levels did not increase in older age^19,20^ or show a U-shaped distribution^3,13,14,21^. Instead, social loneliness showed a marked decrease from late 50’s onwards. These results, therefore, are discrepant with previous studies that have shown that social loneliness gradually decreases from adolescence to young adulthood^29,30^, and then increases in the later stages of adulthood^26^.

We cannot say with certainty what accounts for this discrepancy between past studies and our findings nor can we say what factors are driving the pattern of social loneliness across age in our data. Cross-sectional data is simply not the right kind of data to make those kinds of claims^50^. Instead, we see the value of the current work being the comprehensive description of the pattern of different types of loneliness across age, which has been replicated in two large, nationally representative samples. Moreover, such value was only made possible by making the distinction between loneliness types and by having a sample that is sufficiently large to span the entire adult age range, which past work has not done. Past work tended to focus on age groups, such as groups of younger or older individuals, but not both (e.g.,^26,29,30^), which means that comparisons between age groups were not possible.

At this stage of understanding, therefore, we consider the comprehensive nature of our data and the somewhat discrepant results with past studies to be fertile ground for future research to assess or generate different hypotheses regarding social loneliness, which may have more causal or mechanistic foundations, and then test them using appropriate methods. For example, a prominent hypothesis that could explain the relationship between social loneliness and age is the socioemotional selectivity theory^51,52^. According to this view, physical limitations (e.g., reduced mobility, health issues) that typically decrease the possibility of extensive socialising make older adults prioritise the quality over the quantity of social relationships, which could act as a protective factor for social loneliness. In other words, according to this theory, older adults prioritize fewer but more meaningful relationships, which decreases the risk of social loneliness as maintaining a wide network of social connections is no longer a priority. Further studies could assess this and other hypotheses to get a better understanding of the experience of social loneliness in later life.

### Implications for interventions

The findings from the current study could also help to guide the design of loneliness interventions. In the past, ignoring variation in different types of loneliness across the lifespan might have been one reason for the limited effectiveness of previous loneliness interventions^53–55^. For instance, based on the current findings, middle-aged individuals are much more likely to experience social than emotional loneliness, and could thus benefit more from interventions that specifically tackle social loneliness (e.g., participating in social activities as part of a group^56^), as opposed to interventions that treat loneliness as a unidimensional construct. Based on this, future research should examine how interventions that target a specific loneliness subtype might help alleviate loneliness levels across the lifespan.

### Limitations and future directions

Even though the present study provides novel insight into the experience of loneliness across the lifespan, the results face important limitations. As mentioned before, data from the two NSW cohorts are cross-sectional, which makes it hard to determine causal relationships between age and social and emotional loneliness^50^. Indeed, it is unclear whether the trends observed in this study are cohort-specific, or if they will be carried on as the younger members of the NSW datasets grow older. Further studies could overcome this issue by acquiring longitudinal data to examine the experience of social and emotional loneliness in a single aging cohort.

Another thing to consider is that the findings only focus on one country. This could be problematic because loneliness levels can vary considerably between different regions^20,21,32^. For example, previous research has demonstrated that Eastern European countries are characterised by a gradual increase of both loneliness and age, whereas Western European countries are associated with a rapid increase in loneliness only after ~ 70 years of age^17^. Additionally, recent findings from 237 countries have shown that people in collectivist countries tend to feel less lonely compared to people in individualistic cultures^57^. Future research should compare findings from different regions to obtain a more holistic view of the severity of social and emotional loneliness across the lifespan in different cultures.

A further potential limitation is the use of the DJGLS^34^ as a measurement of social and emotional loneliness. It has been argued that the distinction between social and emotional loneliness in the DJGLS could be partially driven by the valence of items (that is, three negatively phrased items for emotional loneliness and three positively phrased items for social loneliness), as opposed to two underlying factors that correspond to each loneliness subtype^28,58^. However, any account of our findings needs to explain the pattern of data for each loneliness type as a function of age, rather than just an average difference between loneliness types. And, to our knowledge, there is currently no clear theoretical basis or robust evidence that demonstrates that the way people respond to positively and negatively phrased items (as opposed to the way they experience loneliness) is what changes as a function of age. Thus, although it is possible that the valenced nature of the items could contribute to our findings, such a proposal currently lacks theoretical or empirical justification. To contextualise this point further, this limitation is not specific to the current study, as previous studies that estimated the prevalence of social and emotional loneliness across the lifespan also used scales that dichotomised social and emotional loneliness based on positively or negatively phrased items^26,29,30^. As such, we suggest that the development of a revised scale, which can distinguish between different types of loneliness in a cleaner manner, would be an important avenue for future research.

### Constraints on generality

Simons and colleagues^59^ recently argued that it can be useful to place explicit constraints on the generality of findings, which we do here, in terms of the conceptualisation of “age effects”. We do not take the present results to mean that the experience of loneliness across the lifespan is determined by a “pure” chronological effect related to age. Instead, the “age” factor in our analyses is indicative of a complex set of life events that could underlie the experience of loneliness across the adult lifespan. Indeed, different stages in life might be characterised by different events that can drive levels of social and emotional loneliness (e.g., death of a partner that typically occurs in older age might lead to higher emotional loneliness levels). This is consistent with recent meta-analytic findings suggesting that the severity of loneliness across the lifespan is not an age-specific phenomenon but is driven more by personal experiences that may be associated with age in the general population^23^. We think that future work that further characterises the relationship between age, life experiences and loneliness would be valuable.

## Supplementary Information

Supplementary Information.

## Data Availability

The data used in this work are open and can be freely downloaded from the U.K. Data Service website (https://ukdataservice.ac.uk). Furthermore, in accordance with current open science suggestions^60^, we release R Markdown files that record each stage of our analytical process to aid transparency, data sharing, and reproducibility (https://osf.io/yuw6h/).
