# Supplementary material for: Estimating the prevalence of social and emotional loneliness across the adult lifespan
Source: Sci Rep. 2022 Dec 6;12:21045. doi: 10.1038/s41598-022-24084-x (PMC9727057; doi:10.1038/s41598-022-24084-x)
Supplement: Supplementary file 1 — Supplementary Information. [file 41598_2022_24084_MOESM1_ESM.pdf]

Estimating the Prevalence of Social and Emotional Loneliness across the Adult Lifespan

Aikaterini Manoli, Johanna McCarthy, and Richard Ramsey

Supplementary Information

Supplementary Method

We followed the same general analytical approach that was outlined in the main Data Analysis section to create an exploratory model with additional covariates for each of the two NSW cohorts. The variables were sociodemographic and health factors that are frequently associated with loneliness: gender, number of people in the household, marital status, longstanding illness, and an index of overall deprivation (e.g., in terms of income, education, employment, and crime) (Welsh Index of Multiple Deprivation; WIMD). All covariates were treated as categorical predictors, except for number of people in the household which was treated as a continuous predictor (range: 1-8). Gender was coded as “male” and “female”, with “male” treated as a reference category. Longstanding illness was coded as “healthy” and “ill”, with “healthy” considered as a reference category. Overall deprivation was coded as “most deprived”, “Q2”, “Q3”, “Q4”, “least deprived”, with “most deprived” treated as a reference category. Note that the “Q” prefix refers to quintiles of WIMD scores, with Q1 referring to most deprived 20% of individuals, Q5 to least deprived 20% of individuals, and Q2-4 to deprivation levels in the middle. Lastly, marital status was coded as “single” (never married), “married” and “ended”, with “single” considered as a reference category. The marital status level “ended” was acquired after combining responses “separated”, “divorced” and “widowed” in the NSW questionnaire.

Supplementary Table 1

Demographic Information per NSW Cohort

**NSW Cohort**	2016/17	2017/18
n (%)	8,177 (100)	9,343 (100)
**Gender**		
Male	3,618 (44.25)	4,175 (44.69)
Female	4,559 (55.75)	5,168 (55.31)
**Longstanding Illness**		
No	4,213 (51.52)	4,900 (52.45)
Yes	3,964 (48.48)	4,443 (47.55)
**Number of People in Household**		
Sole occupant	2,412 (29.5)	3,001 (32.12)
2 people	3,184 (38.94)	3,601 (38.54)
3-5 people	2,479 (30.32)	2,601 (27.84)
More than 5 people	102 (1.25)	140 (1.5)
**Marital Status**		

Single (never married)	2,101 (25.69)	2,451 (26.23)
Married	3,830 (46.84)	4,283 (45.84)
Ended (married, separated, divorced)	2,246 (27.47)	2,609 (27.92)
**WIMD Level of Deprivation**		
Q1 (most deprived 20%)	1,231 (15.05)	1,549 (16.58)
Q2	1,432 (17.51)	1,690 (18.09)
Q3	1,861 (22.76)	1,970 (21.09)
Q4	2,068 (25.3)	2,144 (22.95)
Q5 (least deprived 20%)	1,585 (19.38)	1,990 (21.23)

Note. *n* = total sample per variable; parenthesis values are percentages for each variable; WIMD = Welsh Index of Multiple Deprivation; Q1 = 1st Quintile; Q2 = 2nd Quintile; Q3 = 3rd Quintile; Q4 = 4th Quintile; Q5 = 5th Quintile.

Supplementary Table 2

Odds Ratios of Parameters for the Exploratory Models in the 2016/17 and 2017/18 NSW Cohorts

Year	Parameter	Odds Ratio	Lower QI	Upper QI
2016/17	Age	0.75	0.73	0.78
	Age^2	0.91	0.89	0.93
	Loneliness Type	0.52	0.49	0.55
	Age * Loneliness Type	1.15	1.10	1.20
	Age^2 * Loneliness Type	1.22	1.18	1.27
	Female	0.91	0.87	0.95
	Married	0.72	0.68	0.77

	eEded	1.10	1.03	1.17
	Num. People in Household	0.93	0.91	0.95
	Q2	0.92	0.87	0.99
	Q3	0.80	0.75	0.86
	Q4	0.81	0.76	0.87
	Least Deprived	0.77	0.72	0.83
	Ill	1.58	1.51	1.65
2017/18	Age	0.72	0.70	0.73
	Age^2	0.92	0.91	0.94
	Loneliness Type	0.55	0.52	0.58
	Age * Loneliness Type	1.25	1.20	1.30
	Age^2 * Loneliness Type	1.25	1.21	1.30
	Female	0.93	0.90	0.97
	Married	0.70	0.66	0.74
	eEded	1.09	1.02	1.15
	Num. People in Household	0.91	0.89	0.93
	Q2	0.94	0.89	1.00

Q3	0.88	0.83	0.94
Q4	0.80	0.75	0.84
Least Deprived	0.78	0.74	0.83
Ill	1.67	1.61	1.74

Note. Lower CI = lower bound of the 95% Bayesian quantile interval; upper CI = upper bound of the 95% Bayesian quantile interval.

Supplementary Figure 1

Full Model (m3b) Chains for 2016/17 NSW Cohort

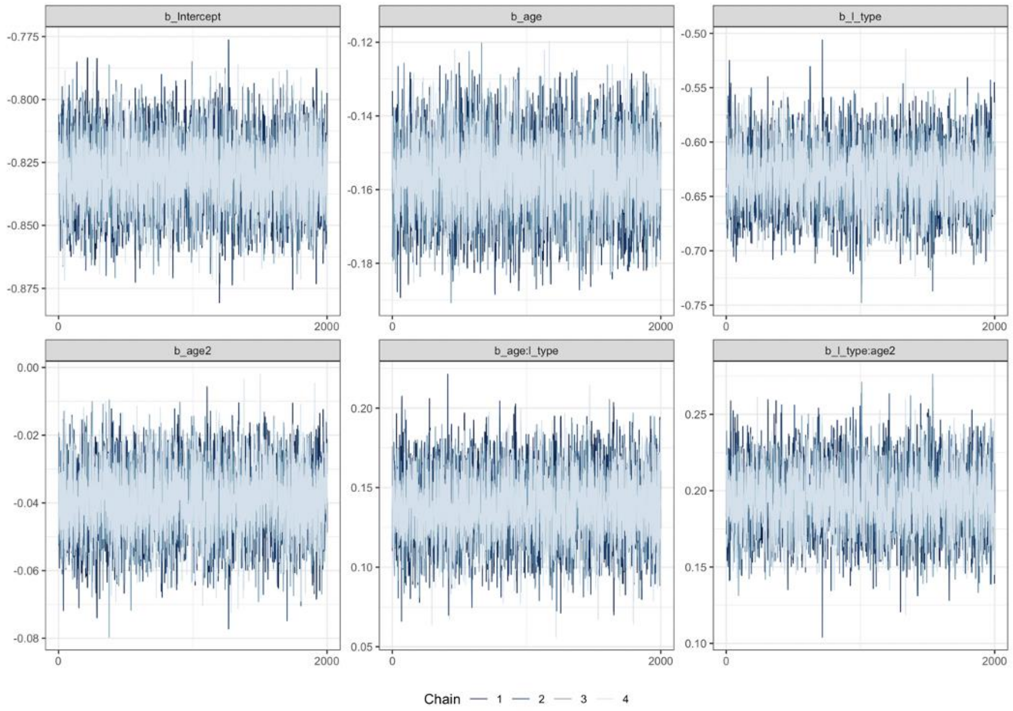

Note. Full model (m3b) chains of parameters intercept, age, loneliness type (l_type), quadratic age (age2) and interaction terms (age:l_type, l_type:age2) for the 2016/17 NSW cohort. The "b_" prefix refers to population-level or fixed effects. All chains seem to have converged reasonably well.

Supplementary Figure 2

Full Model (m3b) Diagnostics for 2016/17 NSW Cohort

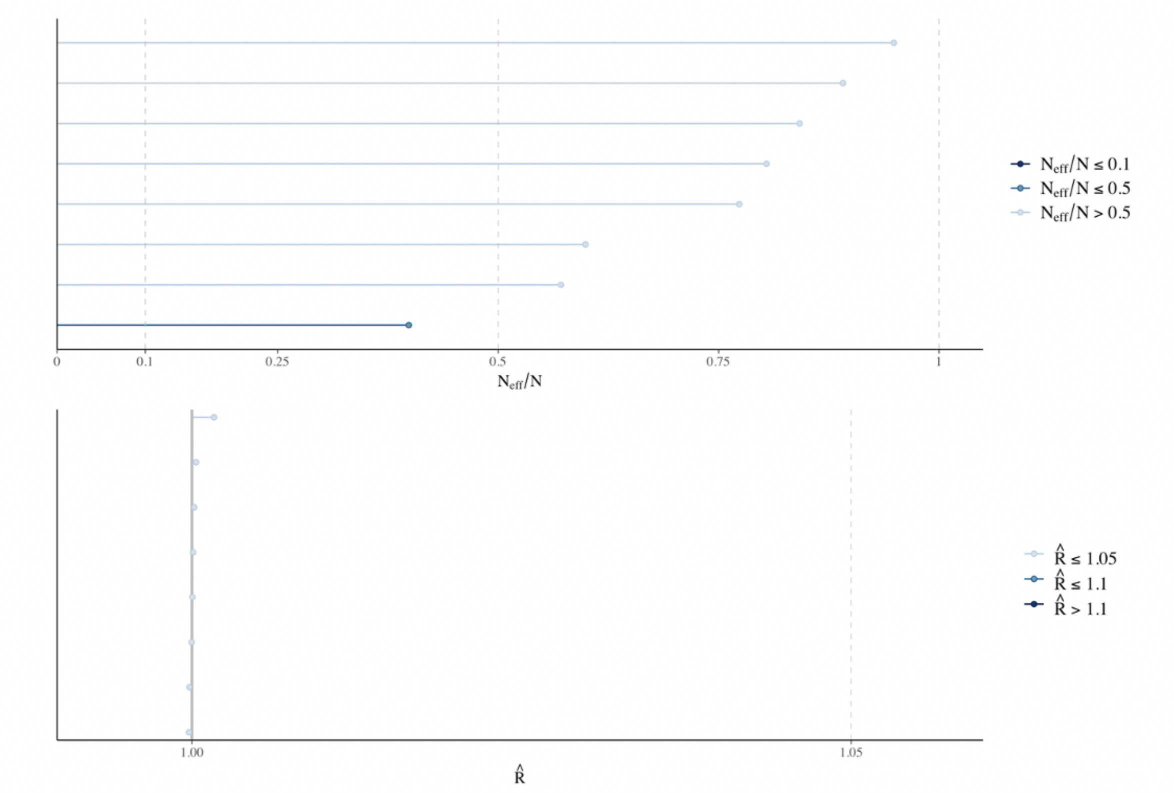

Note. Full model (m3b) diagnostics for the 2016/17 NSW cohort (top graph: N_{eff}, bottom graph: $\hat{R}$). Both model diagnostics are acceptable.

Supplementary Figure 3

Full Model (m3b) Chains for 2017/18 NSW Cohort

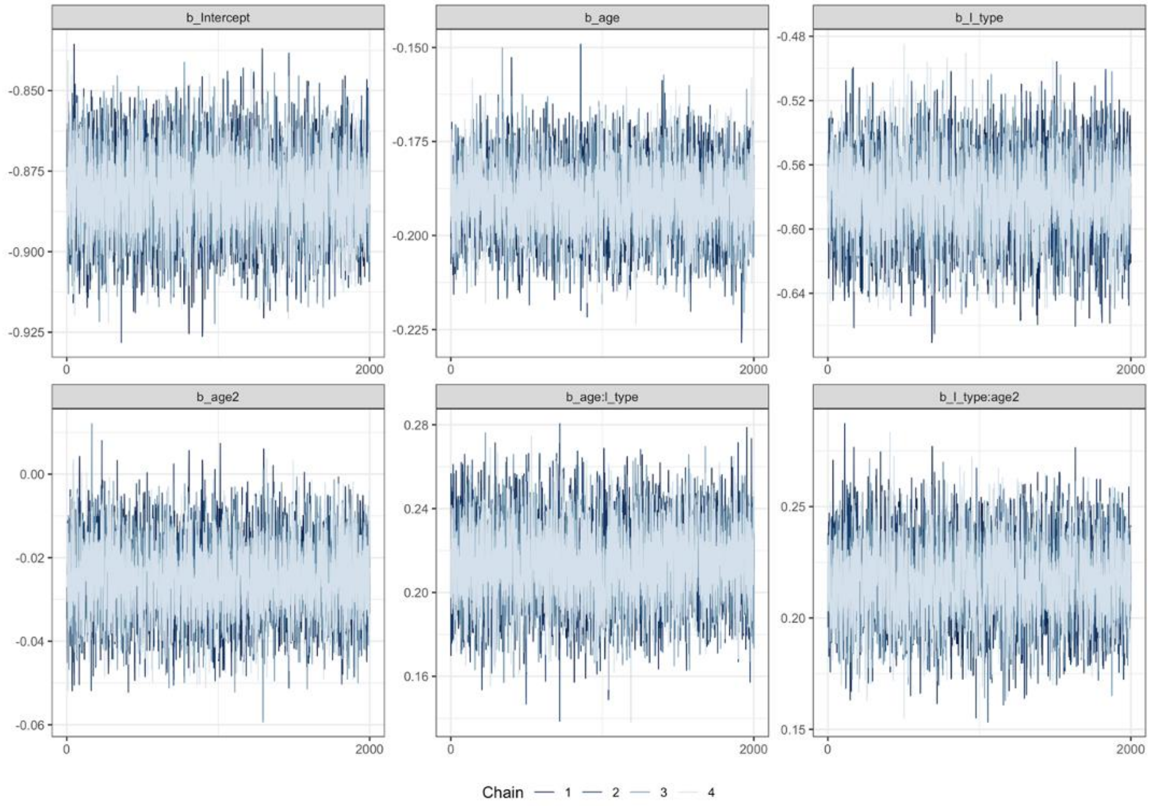

Note. Full model (m3b) chains of parameters intercept, age, loneliness type (l_type), quadratic age (age2) and interaction terms (age:l_type, l_type:age2) for the 2017/18 NSW cohort. . The "b_" prefix refers to population-level or fixed effects. All chains seem to have converged reasonably well.

Supplementary Figure 4

Full Model (m3b) Diagnostics for 2016/17 NSW Cohort

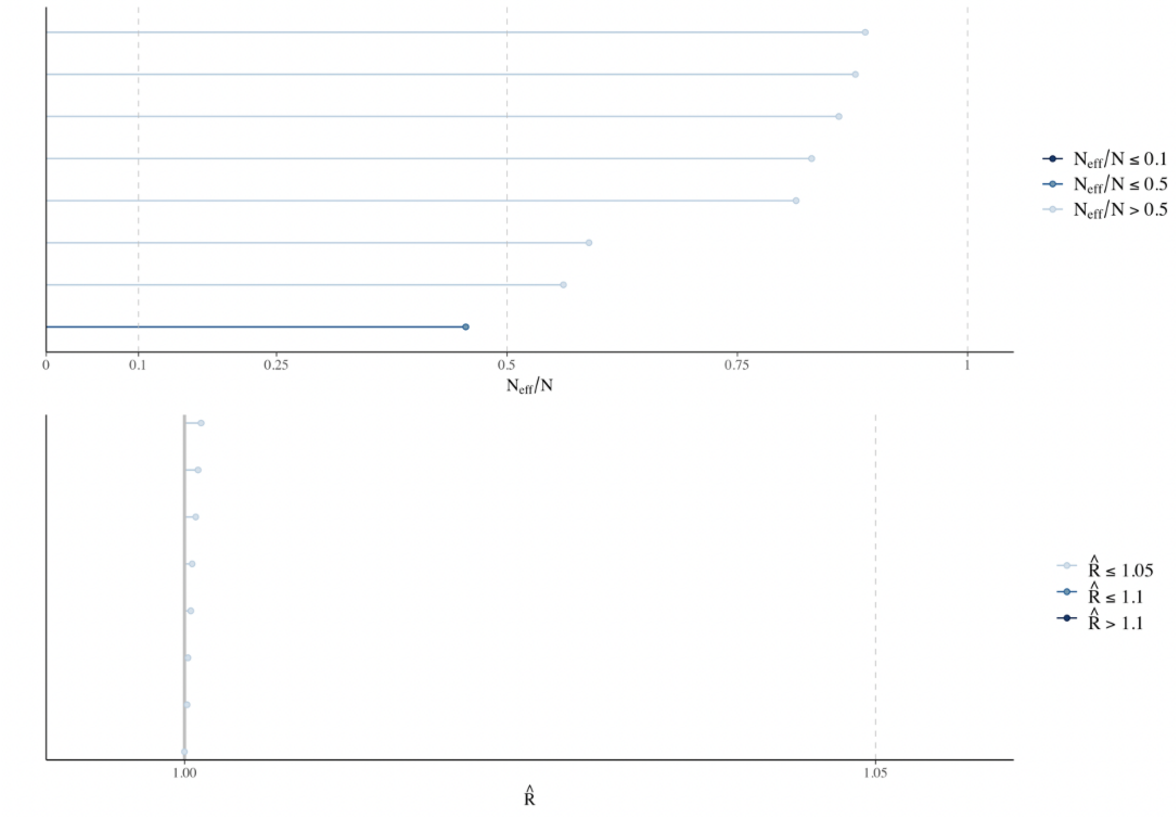

Note. Full model (m3b) diagnostics for the 2017/18 NSW cohort (top graph: N_{eff}, bottom graph: $\hat{R}$). Both model diagnostics are acceptable.

Supplementary Figure 5

Odds Ratios of Parameters for the Full Models with Additional Covariates in the 2016/17 and 2017/18 NSW Cohorts

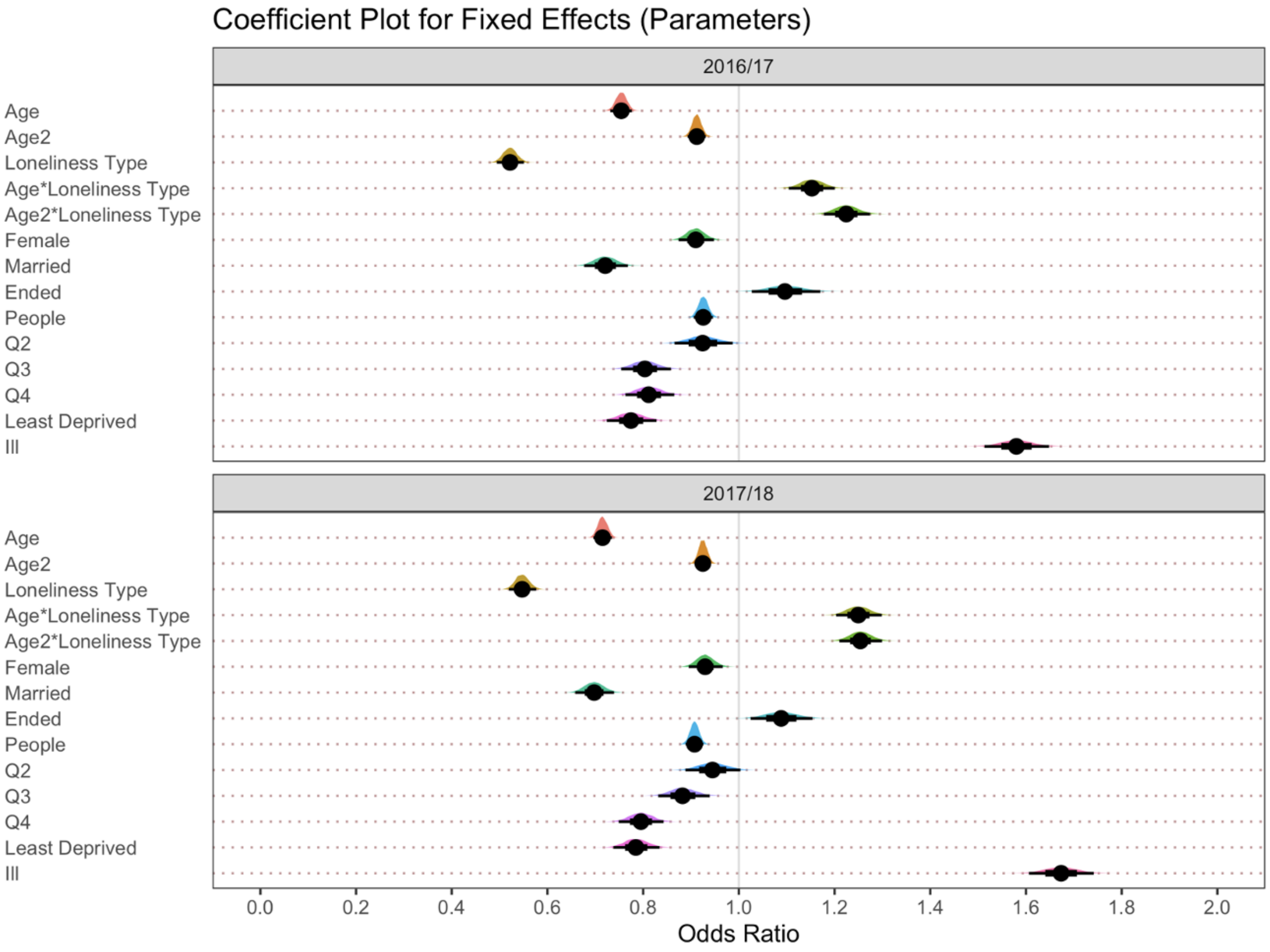

Note. Odds ratios (fixed effects) of parameters for the most complex models with additional covariates (gender, marital status, number of people in the household, overall deprivation, longstanding illness) in each NSW dataset (2016/17, 2017/18). "Female" refers to factor "gender", "married" and "ended" to factor

“marital status”, “people” to factor “number of people in the household”, “Q2-4” and “least deprived” to factor “deprivation”, and “ill” to factor “longstanding illness”.

Supplementary Figure 6

Full Model Predictions for Social and Emotional Loneliness across the Lifespan with Additional Covariates in the 2016/17 and 2017/18 NSW Cohorts

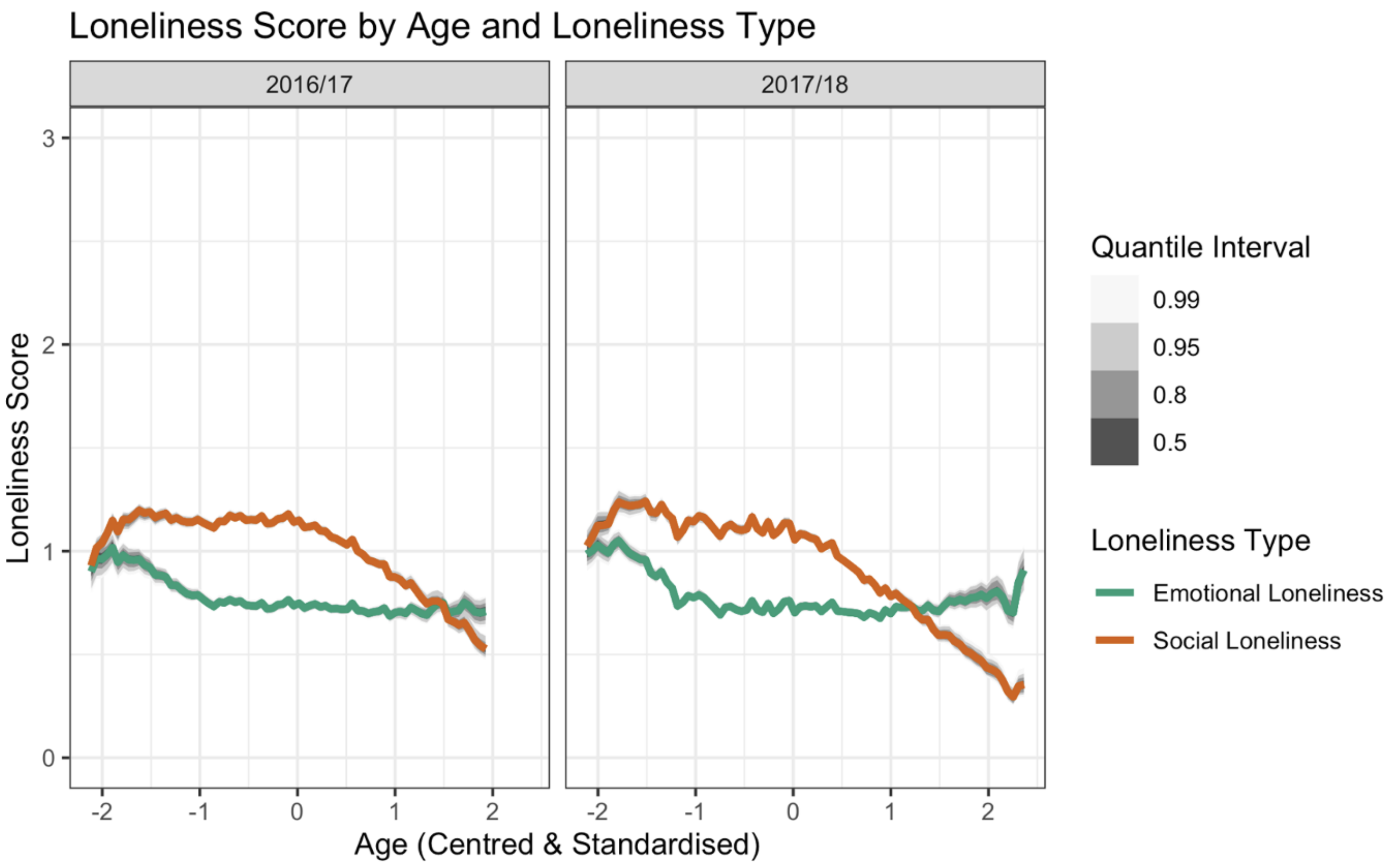

Note. Predictions for social and emotional loneliness scores across the lifespan based on the full model outputs for each NSW cohort (2016/17, 2017/18) with added covariates (gender, marital status, number of people in the household, overall deprivation, longstanding illness), along with 50%, 80%, 95%, and 99% quantile intervals.
